# Evaluating the Performance of Class F Fly Ash Compared to Class G Cement for Hydrocarbon Wells Cementing: An Experimental Investigation

**DOI:** 10.3390/ma17112710

**Published:** 2024-06-03

**Authors:** Youssef Helmy, Sherif Fakher

**Affiliations:** Department of Petroleum and Energy Engineering, The American University in Cairo, New Cairo 11835, Egypt; helmy.youssef@aucegypt.edu

**Keywords:** fly ash, geopolymer cement, Class G cement

## Abstract

The following study presents the results of research in the field of the performance of geopolymers consisting of Class F fly ash with an alkaline activator solution consisting only of sodium metasilicate (Na_2_SiO_3_) and water. The performances of this geopolymer are compared to the those of American Petroleum Institute (API) Class G cement. This comparison is to evaluate the potential of the geopolymer as an alternative to cement in cementing hydrocarbon wells in the oil and gas industry. The gap in the research is determining the performance properties that restrict the use of fly ash in the oil and gas industry. Using only sodium metasilicate as an activator with water, the solution creates a strong binding gel for the geopolymer and activates the aluminosilicate properties of the fly ash. This geopolymer is compared with Class G cement without additives to determine their base performances in high pressure and high temperature conditions, as well as note any properties that are affected in the process. This commences by formulating recipes of these two materials from workable ratios and concentrations. The ratios are narrowed down to the best working models to proceed to comparative performance testing. The tests included exploring their vital performances in fluid loss and thickening time. The results produced suggest that Class G cement generally has less fluid loss at low temperature than the geopolymer but could not maintain its integrity and structure as temperatures increased. Class G cement exhibited stability, consistencies of 100 Bcs (Bearden Consistency Units), and a faster thickening time of 1 h and 48 min when placed under high temperature and high-pressure conditions, respectively. However, the geopolymer showed more consistency regarding fluid loss with respect to rising pressure and temperature, and smoother, less fractured samples emerging from both tests. Though the geopolymer showed stronger performances in thickening and water retention, the experiments showed that it is not a uniform and consistent material like Class G cement. Through the use of different additives and intricate design, the sample may show success, but may prove more difficult and complex to apply than the industry standard and uniform content of Class G cement.

## 1. Introduction

Cement is an essential material in the oil and gas industry, used for constructing oil and gas wells [1]. The main purpose of cement is to maintain wellbore integrity and protect it from hazards such as unwanted formation fluids and pressures, as well as uncontrolled flow of hydrocarbons that could eventually lead to disastrous blowouts. The American Petroleum Institute (API) classifies cement in many types for different purposes, such as depth and environment [2]. The most used class of cement is API Class G cement. Given the environmental, financial, and functional challenges that cement poses for its manufacturing and industrial usage at high temperatures and pressures, researchers and experts in the industry opted to formulate an alternative material that would potentially be more environmentally friendly, less demanding of consumables, and highly efficient at much lower costs [3]. This potential alternative is geopolymer cement using Class F Fly Ash [4]. Fly ash is a pozzolanic aluminosilicate material that is the residue of coal combustion, mainly at power plants and other facilities using coals [5,6]. According to Crook [5], fly ash typically has a lower specific gravity than Class G cement and improves in compressive strength with curing time [7]. Depending on the cement design, some pozzolanic material can decrease permeability when exposed to certain binders, which reduces the potential for damage from corrosive and hazardous substances such as sulphate.

Fly ash classification depends on the institute and its respective codes, based on the Bureau of Standards [8]. According to Code IS 3812-2003-I of the Bureau of Indian Standards, Grade I fly ash is that which results from bituminous coal containing a minimum fraction of silicon dioxide, aluminum oxide, and iron oxide (SiO_2_ + Al_2_O_3_ + Fe_2_O_3_) by mass of 70%. Grade II fly ash results from lignite coal with a minimum fraction by mass of the same compound by 50% [9]. The American Society for Testing and Materials (ASTM) classifies fly ash using a slightly different criterion. According to Code ASTM C618, fly ash may be referred to as either Class C or Class F. Class C is the residue of either combusted lignite or sub bituminous coals with a calcium oxide (CaO) content above 10% or even 20% [10]. Class F fly ash is the residue of either combusted bituminous or anthracite coals with a CaO content below 10% [11]. High calcium fly ash tends to have a reddish appearance, whereas low calcium fly ash is greyish [12]. Fly ash may also be classified based on temperature. Low temperature (LT) fly ash is the result of combustion of coal below 900 °C. High temperature (HT) fly ash is the residue of coal combusted below 1000 °C. In the oil and gas industry, it is used as a supplementary material to make lightweight slurries as part of oil and gas cementation [7]. This is in part due to the practice of recycling waste to manufacture brick and cement products while saving energy, resources, and costs and reducing carbon emissions. However, fly ash faces challenges in proportion, compatibility with other materials, and consistency in the quality of the material, for which it can display varying performances [12]. When used as a sole product, fly ash is prevalently used and explored in producing geopolymers [8].

Unlike Class G cement, geopolymers are inorganic binders that are the result of mixing its aluminosilicates, like fly ash from coal-fired power plants, with an alkaline solution [13]. Several researchers indicate that the typical alkaline solution consists of mixing alkali activators, such as sodium silicate (Na_2_SiO_3_) and sodium hydroxide (NaOH), with water [1,5,13]. The reaction of mixing the geopolymer with the solution results in the formation of an aluminosilicate gel that binds the fine-grained slurry into a solid [14]. The concept of using geopolymers as an alternative to cement has become increasingly popular due to the potential of reducing waste by recycling, repurposing, and reusing it in the same function as a building material with strong and efficient mechanical, chemical, thermal, and durability properties compared to those of Class G cement [15]. Researchers are more inclined to use fly ash as the aluminosilicate base for geopolymers [4]. According to Zain et al., fly ash contains the most sufficient composition of silicone and aluminum and is a reusable waste that does not require natural resources that have a higher priority in being essential for human life [16]. Fly ash is rated at variable market prices but is generally cheaper than Class G cement and can even be obtained, at times, at very low cost [17,18]. Yu et al. found that anhydrous sodium metasilicate (ASM) has the potential to be used as an alkaline activator for the geopolymer alone rather than with sodium hydroxide. The activator provided an enhanced strength to the sample over long periods compared to the sample with sodium hydroxide [19]. Haruna et al. add that the densities of the anhydrous sodium metasilicate solutions increases with the increase of the activator content [20]. In reaction to drilling fluids, Class F fly ash-based geopolymers experienced lower fluid loss than Class G cement, greater retention of compressive strength and durability, and lower viscosity, which indicates better injectivity [21,22]. Additional additives such as superplasticizers and hematite have been found to aid in preventing corrosion, controlling the thickening time, and improving the overall performance of the geopolymer in well environments [23,24,25,26].

While the use of Class F fly ash geopolymers as a substitute of Class G cement is theoretically and experimentally accepted by previous researchers, it is not applied in any field operations. A major issue noted by Adjei is that fly ash geopolymer has a high reactivity to water-based mud, which accelerates the development of the aluminosilicate gels [23]. Salehi notes that the thickening time of geopolymers is typically shorter than that of Class G cement. The time can be shortened or extended depending on multiple factors, such as temperature and additive types and concentrations in the slurry. In his experiments, Salehi used superplasticizers and retarders to regulate the thickening time. Through testing, he found that the compressive strength of the geopolymer was generally higher than cement over a long curing period. Furthermore, the higher the temperature and the ratio of sodium silicate to sodium hydroxide, the higher the compressive strength. Salehi finds that the key challenge in applying fly ash based geopolymers into industrial usage is the short thickening time, and volatile reactions to temperature [24,25]. This paper focuses on evaluating the performance of the geopolymer cement comprising of Class F fly ash compared to Class G cement in the cementation of oil and gas wells, particularly regarding fluid loss and thickening time. The process involves formulating several concentrations of fly ash, water, and a single alkaline activator, sodium metasilicate (Na_2_SiO_3_). The purpose of only using Na_2_SiO_3_ as an alkaline activator is that it is the solid and more concentrated form of sodium silicate; it allows us to discard the usage of sodium hydroxide (NaOH), which is dangerous to handle. The formulations were made through a series of workable ranges from previous works, in which the formulations are then put into practicality by using laboratory utilities to mix the ingredient materials at the required concentrations with specific mixing procedures, and then poured to set and harden in batches to later examine. The most efficient and most well-set formulation samples are then used as references for later testing to compare results with Class G cement samples.

## 2. Experimental Description

### 2.1. Experiments Methodology

The methodology for this study’s innovative experimentation involves following the experimental approaches and methodologies of previous researchers [1,4,13,16,18,19,20,21,22,23,24,25,26]. This includes mixing batches of geopolymer consisting of Class F fly ash with the required alkali activator solution, in this case, sodium metasilicate, followed by mixing batches of Class G cement with only the base cement and water. The materials are mixed as such to test their respective performances under the experiment conditions and types with no involvement of additives, as with the Class G cement, and as little activating substances as possible, as with the geopolymer.

The API Class G cement’s design follows API mixing materials and procedures. The geopolymer’s design follows in the steps of previous researchers regarding workability ratios and concentrations of water, fly ash, and sodium metasilicate. The geopolymers are then optimized to set and harden when curing.

Following the attainment of a formulation of a working geopolymer slurry, the slurries of both materials are tested using Fluid Loss and Thickening Time experiments. The Fluid Loss experiment aims to reflect the materials’ ability to retain water and maintain their structures and integrity under high pressure and temperature conditions around the well. The Thickening Time experiment aims to reflect the materials’ injectivity, thickening time, setting, and integrity around the well under high temperature and pressure conditions.

### 2.2. Experiment Material

The material used to conduct the experiments is as follows:

- *Fly Ash (SiO_2_ + Al_2_O_3_ + Fe_2_O_3_):* This experiment consists of Class F Fly Ash; as such, the fly ash being used mainly consists of silica, alumina, iron oxide, and a low lime/calcium and content.
- *Sodium Metasilicate (Na_2_SiO_3_):* Sodium metasilicate is the solid and more concentrated form of liquid sodium silicate. This concentration provides a strong alkaline activation of the silicates in the fly ash.
- *API Class G Cement:* The cement used in this experiment is API Class G cement, the most commonly used cement type in completing the construction of oil and gas wells. This material mainly consists of high lime and silica content, as well as iron oxide and alumina.
- *Water:* The water used for this experiment was at room temperature tap water. This water was used to mix the different cements.

### 2.3. Geopolymer Formulation and Procedure

#### 2.3.1. Geopolymer Initial Formulation Calculations

To formulate the geopolymer slurry samples, the workability ranges found by previous researchers [1,4,13,16,18,19,20,21,22,23,24,25,26], shown in Table 1, are presented:

Based on the findings and trials of previous researchers [1,4,13,16,18,19,20,21,22,23,24,25,26], Table 1 shows the experimental concentrations of water in the workable range for mixing the alkaline solution, as well as the workable ratios between the weight of the fly ash and the weight of the alkaline activator/binder.

(1) $$Binder\;\left(g\right)=\frac{Fly\;Ash\;(g)}{Binder\;Ratio}$$

By dividing a uniform weight for fly ash by each ratio, as seen in Equation (1), the weights of the sodium metasilicate were determined for each ratio, as shown in Table 2:

Using the five weights for sodium metasilicate, determined in Table 2, each weight sample was mixed alongside one of the seven concentrations of water seen in Table 1, relative to the total weight of the slurry. This led to 35 formulations of alkaline solution; each sample was mixed with 170 g of fly ash. The best set sample and formulation proceeded to the experiments.

The determined weights of each geopolymer sample’s sodium metasilicate are then used alongside the weight of the fly ash in Equation (2) as part of a sum of the geopolymer’s solid weight. That sum is divided by the solid composition of the geopolymer, which is determined by subtracting the water composition of the geopolymer. The result is the total weight of that particular geopolymer sample.

(2) $$Total\;\left(g\right)=\frac{Fly\;Ash\;\left(g\right)+Binder\;(g)}{1-Water\;(\%)}$$

Equation (3) was used; the total weight subtracted the sum of the fly ash weight and the sodium metasilicate weight to determine the water weight mixed with each geopolymer sample.

(3) $$Water\;\left(g\right)=Total\;\left(g\right)-Binder\;\left(g\right)-Fly\;Ash\;(g)$$

To follow potential patterns to further explain and analyze the reactions of each geopolymer sample and to fill any missing values, as well as assist in potentially optimizing the formulations, the following equations are implemented:

(4) $$Binder\;(\%)=\frac{Binder\;(g)}{Total\;(g)}$$

Equation (4) determines the composition of the sodium metasilicate by dividing its determined weight by the total weight of that geopolymer sample.

(5) $$Fly\;Ash\;(\%)=\frac{Fly\;Ash\;(g)}{Total\;(g)}$$

Equation (5) also determines the composition of the fly ash by dividing the uniform weight by the determined total weight of that geopolymer sample.

Densities are not prioritized in the formulations, as the main objective of the study is to test the performance of the geopolymer’s fluid loss and thickening time capabilities regardless of the weight difference compared to Class G cement.

#### 2.3.2. Geopolymer Mixing Procedure

**Figure 1:**
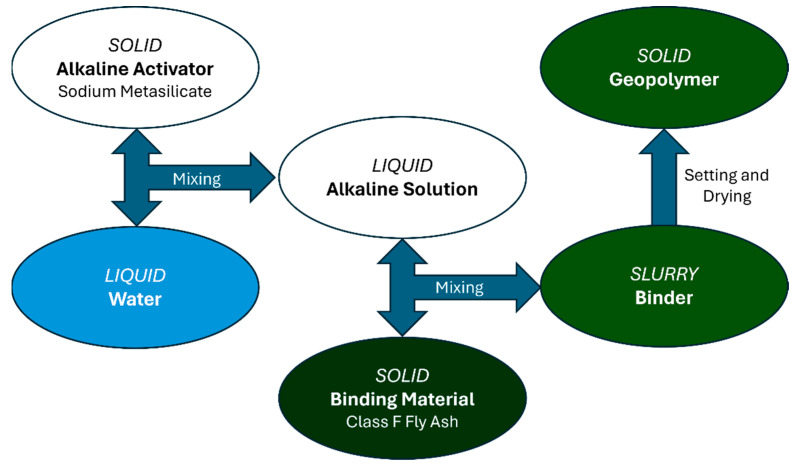
Geopolymer Mixing Procedure.

As demonstrated by Figure 1, for each of the 35 formulations, the water was first mixed with the sodium metasilicate for 1 min at 2000 RPM to form the alkaline activator solution, after which the fly ash was gradually added to the solution to avoid clumping and mixed for 4 min at the same speed. After the mixing time, the slurry was poured into trays to allow the slurry to set.

#### 2.3.3. Optimization of Geopolymer Samples

Following the mixing and setting of the initial 35 samples, it was observed that the samples with a fly ash/binder ratio of 15 and 20 set best and maintained their form and hardness without crumbling or deteriorating like the other samples, particularly those with a water concentration of 20%. It is also worth noting that the samples with a ratio of 15 had a sodium metasilicate content range from 3.75% to 5.63% and that the samples with a ratio of 20 had a sodium metasilicate content ranging from 2.86% to 4.29%. As a result, to optimize the final set of geopolymer samples, a second round of formulations was conducted with the approach of increasing the binder concentration while decreasing the fly ash/binder ratio to strengthen the geopolymer bonding and hardening. As shown in Table 3, the samples use one weight of fly ash. Given that the samples with 20% water concentration set best initially, the second round only used 20% water concentration.

The next step includes a set of sodium metasilicate concentrations (10%, 12%, 15%, 17%, and 20%). In total, five test samples were produced to determine the finalized batch of geopolymers that proceeded to testing.

(6) $$Fly\;Ash\;\left(\%\right)=1-Binder\;\left(\%\right)-Water\;(\%)$$

Using Equation (6), the content of the fly ash is determined by subtracting the geopolymer’s respective concentration of the sodium metasilicate and the 20% water concentration from 100%.

(7) $$Total\;\left(g\right)=\frac{Fly\;Ash\;\left(g\right)}{Fly\;Ash\;(\%)}$$

Equation (7) is then used to determine the total weight of each of the five geopolymer samples by dividing their established fly ash weight of 170 g by their respective fly ash composition determined in Equation (6).

Equation (8) multiplies the binder test concentration in Table 3 with the total geopolymer weight to determine the binder weight of that sample. Equation (9) multiplies the water concentration of 20% with the total weight of that geopolymer sample to determine its water weight in the mix. The fly ash weights of 170 g are then divided by the binder weights found in Equation (8) to determine the fly ash/binder ratio of each sample, as shown in Equation (10).

(8) $$Binder\;\left(g\right)=Binder\;\left(\%\right)*Total\;(g)$$

(9) $$Water\;\left(g\right)=Water\;\left(\%\right)*Total\;(g)$$

(10) $$\frac{FA}{SS}Ratio=\frac{Fly\;Ash\;\left(g\right)}{Binder\;(g)}$$

The final data of compositions and weights for all five samples can be seen in Table 4, with which the mixing procedure is repeated.

### 2.4. Cement Formulation and Procedure

#### 2.4.1. Cement Formulation Calculations

To prepare a cement sample for which a comparative analysis could be conducted, samples of a single cement recipe were used that consisted of Class G cement and water, according to API standards [27]. To prepare this type of cement to those standards, the water weight was set at 44% of the base Class G cement weight. To be equivalent to the geopolymer mix in terms of composition for comparison, one sample of this cement would require 170 g of Class G cement and 74.8 g of water.

#### 2.4.2. Class G Cement Material and Mixing Procedure

Much like the geopolymer procedure, water is poured into the mixer (Constant Speed Mixer Model 686CS Fann, Houston, TX, USA) first and is mixed at 2000 RPM. The Class G cement is then gradually added to the mix to avoid clumping and to keep the slurry smooth. Once the cement is completely added, the slurry is to continue mixing for 4 min. Once the time ends, the slurry is poured into trays to set and later be tested.

### 2.5. Experimental Scope

Once the geopolymer and cement samples are prepared, a set of experiments are conducted on them to compare their performances and results with regard to slurry setting and stability, fluid loss, and thickening time.

#### 2.5.1. Fluid Loss Test Procedure

**Figure 2:**
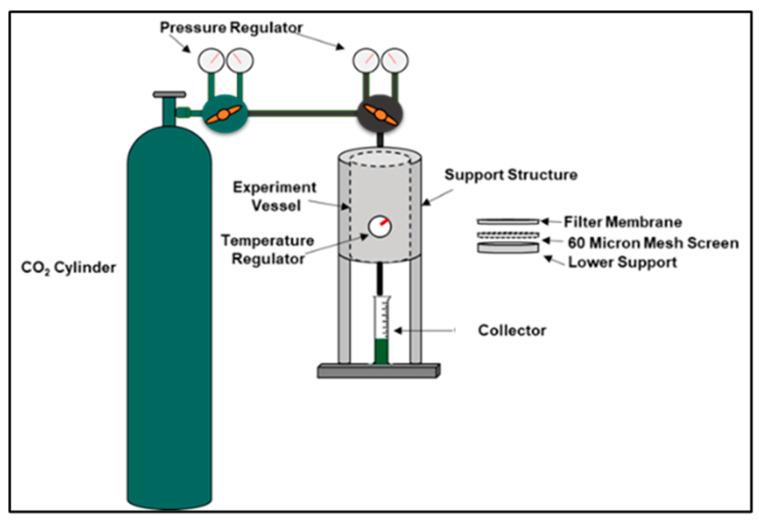
Fluid Loss Test Procedure.

The Fluid Loss Test, shown in Figure 2, was conducted on both the geopolymer and Class G sample slurries. They were poured into a vessel and were placed under 1000 psi with a back pressure of 200 psi at temperatures of 20 °C, 60 °C, and 100 °C for 30 min each to determine each sample’s ability to retain water. The ability of a slurry to retain water under these conditions is measured by draining the sample’s water volume in a graduated cylinder.

#### 2.5.2. Thickening Time Test Procedure

**Figure 3:**
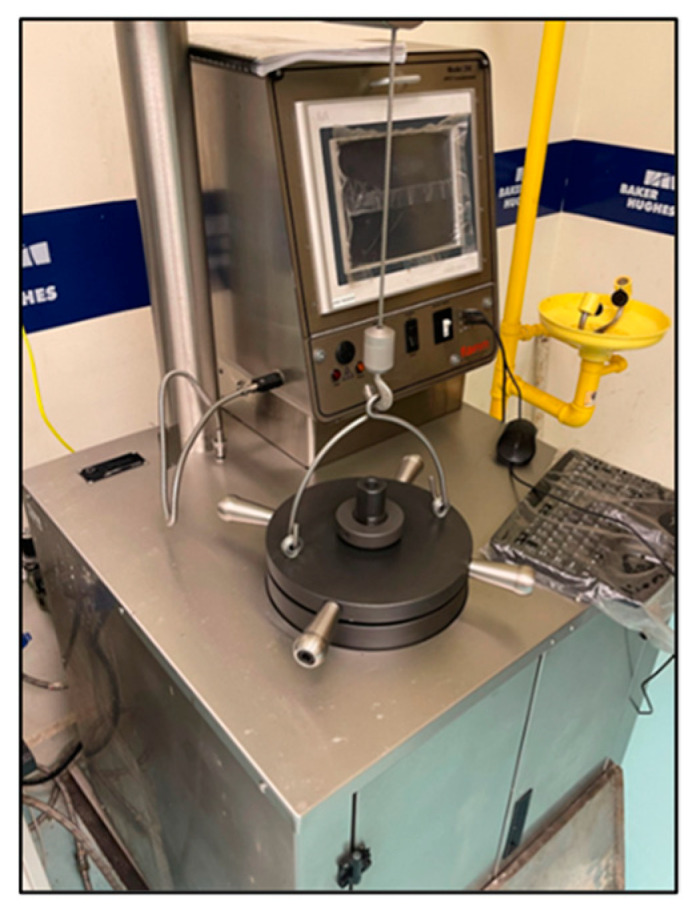
Consistometer For Thickening Time Test.

The Thickening Time Test is conducted on each slurry by pouring it into a chamber that is placed in a consistometer, shown in Figure 3. The chamber is subjected to an ambient pressure of 1000 psi and temperatures of 20 °C, 60 °C, and 100 °C until the slurry reaches a consistency of 100 Bc (Bearden Consistency Unit), which indicates it has completely thickened. The test times how long it takes for the given cement slurry to thicken under said conditions, how stable the cement is after the test is completed under those consistent conditions, as well as other rheological properties during that time.

## 3. Results and Discussion

The results produced from the previously mentioned experiments were gathered, summarized, and analyzed to explain the behavior of both the geopolymer and cement samples under certain tests and conditions. The results include observations of the setting of the cement and geopolymer slurries into samples and tracking their curing over time. Once the samples hardened, their respective recipes were repeated to concoct slurries that were tested for fluid loss and thickening time.

### 3.1. Geopolymer Slurry Setting

**Figure 4:**
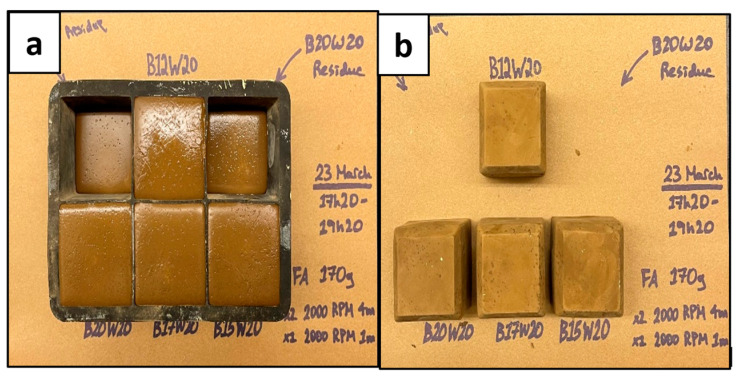
Geopolymer Samples; Wet (**a**) and Dry (**b**).

**Figure 5:**
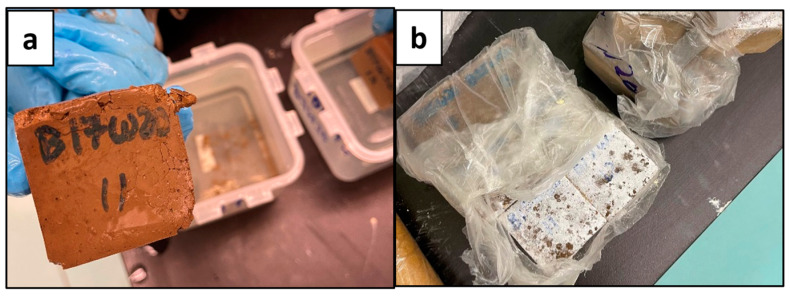
Deteriorated Geopolymer Samples; B17W20 (**a**) and B20W20 (**b**).

The setting of the geopolymer slurry into a solid sample is very important for the purpose of this experiment, as it is meant to resemble a hardened cement sample in the annulus of a wellbore. The geopolymer slurries were poured into trays to set and produce cube samples to cure over 7 days (as shown in Figure 4 and Figure 5) to determine the best working sample from the different batches of concentrations and ratios previously mentioned. As Crook mentions, the alumina/silica content of Class F fly ash is not uniform and varies from batch to batch [7]. Therefore, this geopolymer had to be specially designed through trials of multiple mixes of different ratios and concentrations of its material content to set well according to its batch of Class F fly ash. In industrial applications, this would be complex, time consuming, and require a method of determining the chemical composition of the fly ash to determine the appropriate activator(s) and their concentrations, which would be costly.

The results shown in Figure 4 and Figure 5 indicated that the geopolymers with a sodium metasilicate content of 12% (B12W20) and 15% (B15W20) set best; these were used for the experimental phase. The sample with 10% content (B10W20) was not strong enough and did not set well, and the samples with 17% (B17W20) and 20% content (B20W20) eventually deteriorated during the seven day curing period.

### 3.2. Cement Slurry Setting

**Figure 6:**
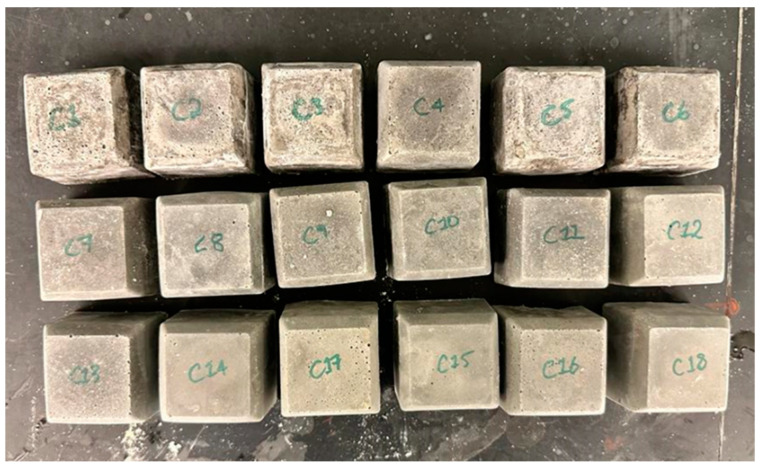
API Class G Cement Dried Samples.

The cement slurry setting is equally as important, as it is meant to be the baseline sample the geopolymer will be tested against, without any additives. The cement slurry was mixed in accordance with API standards for mixing Class G cement by mixing the Class G cement aggregates with water at a weight of 44% of the weight of the aggregates [2]. This was followed by pouring the slurry into trays to set and produce cube samples to similarly cure for seven days (as shown in Figure 6). Given that Class G cement possesses uniform material contents and reactions with each batch, only one slurry recipe was needed. This type of cement is commonly used in the oil and gas industry as a standard, as it is simple to mix, can be altered with certain additives, and consumes less time.

The observations in Figure 6 showed that the samples were much firmer than those of the geopolymer samples. The one Class G cement cube was more difficult to deteriorate and did not get crushed by the squeeze of a hand, unlike the geopolymer cubes.

### 3.3. Fluid Loss Test

**Figure 7:**
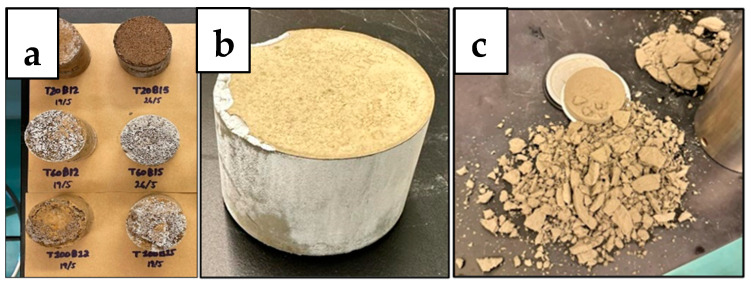
Fluid Loss Test Residue of Geopolymer (**a**), Cement T20 (**b**), and Cement T60 (**c**).

Once the desired samples have hardened, their respective recipes are repeated to produce slurries to be tested for fluid loss. As mentioned by Ahdaya, the purpose of the experiment is to test the samples’ ability to retain water and maintain their structure under specific temperature and pressure conditions [4]. This test simulates the setting of the geopolymer slurry and the setting of the Class G cement slurry as the cement used in the annulus of a wellbore under high pressure and high temperature conditions. As shown in Figure 7a,b, certain samples maintained their structure and produced water in a graduated cylinder under 30 min, while other samples, such as Figure 7c, broke down without producing water, as it quickly evaporated.

**Figure 8:**
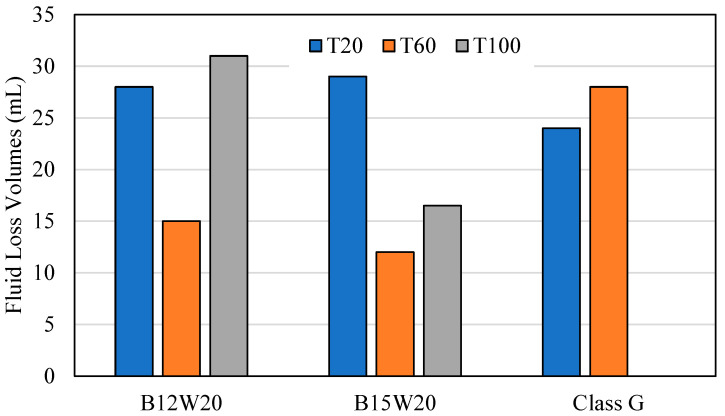
Fluid Loss Volumes.

The fluid loss test was conducted at 1000 PSI at 20 °C, 60 °C, and 100 °C for the geopolymer samples B12W20 and B15W20 and for the Class G cement sample. Figure 7 expresses how the slurry for each test batch came out of the test chamber after each test. Figure 8 expresses the fluid lost in milliliters (mL) from each sample at each temperature.

At 20 °C, the geopolymer samples had a relatively high level of fluid lost, between 28 and 29 mL, and the samples came out of the fluid loss tester in a smooth cylinder shape. In comparison, the cement sample had less fluid lost at 24 mL, with a relatively smooth cylinder shape; however, it had edges that were chipped off that the top.

At 60 °C, the geopolymer samples had less fluid loss than before, ranging between 12 and 15 mL, and the samples were taken out of the tester intact but showing signs of slight decalcification. The cement sample, however, produced 28 mL of fluid lost, this time with a higher difference to the geopolymer, coming out almost completely crumbled due to the high pressure, high temperature conditions.

At 100 °C, B15W20 showed a fluid loss of 16.5 mL, while B12W20 showed a fluid loss of 31 mL. Both samples emerged from the tester with even greater signs of deterioration due to the high temperature and high pressure conditions. The cement sample came out of the chamber completely deteriorated and evaporated the fluid within.

The Class G cement showed less fluid loss than fly ash and a quicker thickening time, with a good integrity at high temperatures and pressures. Further additives can be provided for the cement design to strengthen the bonding, extend the slurry, and control the thickening time.

### 3.4. Thickening Time Test

**Figure 9:**
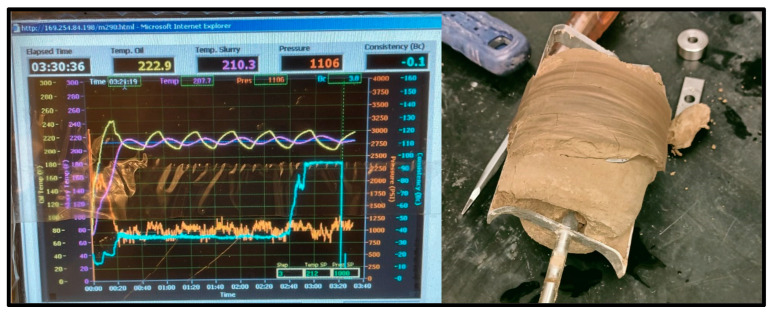
100 °C Geopolymer sample consistometer readings and thickened result.

**Figure 10:**
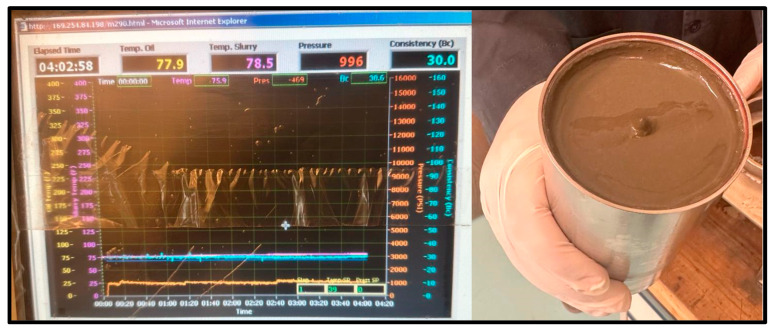
20 °C Cement sample consistometer readings and slurry result.

**Figure 11:**
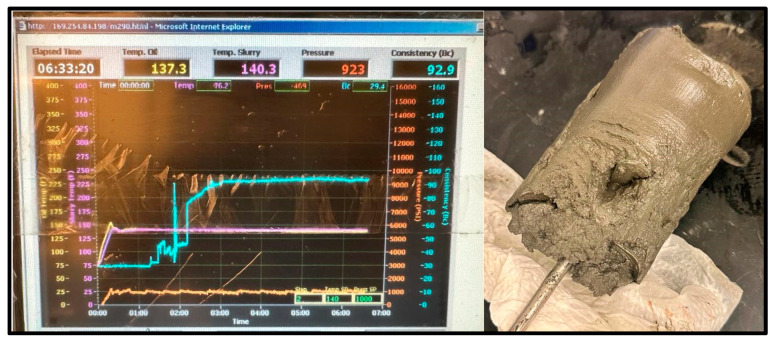
60 °C Cement sample consistometer readings and slurry result.

**Figure 12:**
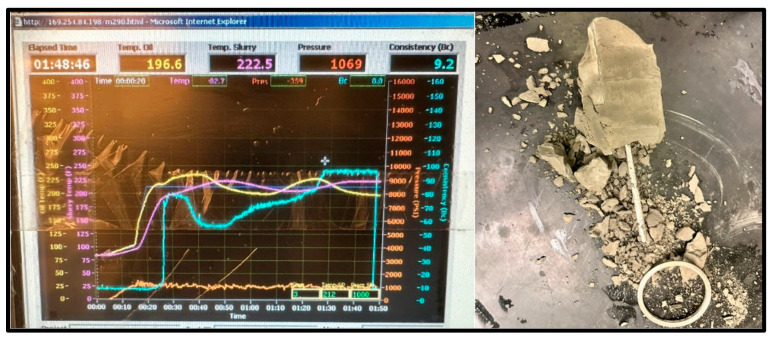
100 °C Cement sample consistometer readings and slurry result.

Thickening time is a crucial parameter to be measured between these materials. This parameter determines the time needed for a cementing material to thicken and set in the wellbore for the operation required. Both samples’ working slurries were poured into a chamber, then sealed and placed into a HTHP consistometer. Using the consistometer’s computer and software (Fann M290 Consistometer M290 HPHT Consistometer Interface (Version 1)), the temperature and pressure conditions were set, and the tests commenced. The consistometer exposed the sample slurries to the set temperature and pressure conditions to resemble downhole conditions and tracked the samples’ consistency against the time that passed. The tests were conducted for the geopolymer (shown in Figure 9) and the cement (shown in Figure 10, Figure 11 and Figure 12) until the samples reached a consistency of 100 Bcs, or until a limit of 8 h. Suppiah explains the significance of testing the rheological properties of a cementing material, as it provides a perspective into the pumpability of the material into the annulus and what additives may be required to improve it [1].

The thickening time test was conducted at 1000 PSI at 20 °C, 60 °C, and 100 °C for the geopolymer samples and the Class G cement sample. The geopolymer sample remained at a consistency of 17 Bc for over 8 h on both the 20 °C and 60 °C tests with the slurries coming out unhardened, indicating that its thickening time was too slow. On the other hand, the 100 °C test reached 100 Bc in just over 3 h and 30 min, shown in Figure 9.

As for the cement sample, the slurry was poured into the consistometer for the same three temperatures and pressure conditions. For the 20 °C test, the sample maintained a consistency of 30 Bc after 4 h and remained as a slurry, as shown in Figure 10.

The cement sample for the 60 °C test reached a peak consistency of 92.9 Bc after 6 h and 33 min; it set more than the 20 °C sample, maintaining the cylinder shape of the consistometer’s chamber, but did not harden, as shown in Figure 11.

The cement sample for the 100 °C test reached the maximum consistency of 100 Bc after 1 h and 48 min and completely set, as shown in Figure 12; however, the sample showed major signs of deterioration.

The Class G cement showed more consistency regarding its natural setting properties without additives, whereas Class F fly ash would, by default, require an additional activator to strengthen the bonding, and that activator would require thorough design to fit the aluminosilicate and calcium properties of the fly ash. The lack of NaOH could potentially have affected the setting, integrity, and durability of the geopolymers since it is a potentially balancing factor in the aluminosilicate gel formed within the geopolymer. This is because sodium provides an increase in compressive strength.

## 4. Conclusions

Given the findings provided by previous researchers as well as the results and observations from the experiments conducted in the laboratory, Class F fly ash geopolymers face several complex challenges to implement them in the oil and gas industry as alternatives to API Class G cement. While its experimental properties have shown performances that exceed that of Class G cement, it would require more additives for use in high temperature and high pressure environments. Upon planning for further testing and acquiring a further batch of the same Class F fly ash for the geopolymer from the same supplier, the geopolymerization of the slurry did not set as the previous batch did; thus, further experiments could not be completed. Upon further research into this matter, it was later indicated by Crook et al. in the Petroleum Engineering Handbook that aside from Class F fly ash having different source origins such as volcanic ash and residue from coal-fired power plants, industrial batches can vary in chemical content, and thus affect the geopolymer’s performance such as thickening time, mechanical properties, and potential decalcification [7]; furthermore, the ratio and difference in alumina and silica ions can greatly affect the effects of the fly ash with other materials, which can lead to weak setting or deterioration. These findings demonstrate that unlike Class G cement, Class F fly ash geopolymers would not be practical in cementing applications, as each individual batch requires its own intricate design plan for activation and curing, which would make the cementing process much more difficult.

The main findings from this study are as follows:

- There is an inconsistency in the alumina/silica content as well as CaO composition with fly ash batches from different origins, and this results in inconsistent reactions.
- Though Class F fly ash geopolymers are said by researchers to accelerate more than Class G cement in setting, it was found to differ depending on the batch; furthermore, Class G cement thickened at high temperatures and pressures faster than the geopolymer with a high consistency.
- Class G cement showed a more consistent fluid loss with the increase of temperature and pressure than Class F fly ash and can have additives to improve its integrity.
- Class G cement can be used for water, oil, and synthetic-based drilling fluids, whereas there is little known research of using Class F fly ash with drilling fluids.
- Sodium metasilicate and sodium hydroxide are required for the alkaline activator that the geopolymer needs to have both a strong and balancing aluminosilicate gel that gets stronger with time, water exposure, and temperature.
- Additives such as extenders, retarders, thickeners, dispensers, and more can be added to the cement to have it react in a function according to the cement design and purpose of the operation.

## Figures and Tables

**Table 1:** Initial geopolymer formulation workable ratios and concentrations.

Fly Ash Weight (g)	170						
Fly Ash (g)/Binder (g) Ratio	15	20	25	30	35		
Water Concentrations (%)	10%	15%	20%	25%	30%	35%	40%

**Table 2:** Binder weights for each fly-ash/binder ratio.

Fly Ash Weight (g)	170				
Fly Ash (g)/Binder (g) Ratio	15	20	25	30	35
Binder Weight (g)	11.3	8.5	6.8	5.7	4.9

**Table 3:** Optimized geopolymer formulation ratios and concentrations.

Fly Ash Weight (g)	170				
Binder Concentrations (%)	10	12	15	17	20
Water Concentration (%)	20%				

**Table 4:** Optimized geopolymer formulations.

Serial Number	FA/SS Ratio	Concentration (%)			Weight (g)			
		Fly Ash	Sodium Metasilicate	Water	Fly Ash	Sodium Metasilicate	Water	Total
B10W20	7.0	70%	10%	20%	170	24.29	48.57	243
B12W20	5.7	68%	12%	20%	170	30.00	50.00	250
B15W20	4.3	65%	15%	20%	170	39.23	52.31	262
B17W20	3.7	63%	17%	20%	170	45.87	53.97	270
B20W20	3.0	60%	20%	20%	170	56.67	56.67	283

## Data Availability

Data available on request.
